# Pseudoaneurysm of the anterior tibial artery: A rare complication of proximal tibial steinman pin insertion

**DOI:** 10.4103/0019-5413.77140

**Published:** 2011

**Authors:** Tarun Suri, Vineet Dabas, Sumit Sural, Anil Dhal

**Affiliations:** Department of Orthopedics, Maulana Azad Medical College and Associated Lok Nayak Hospital, New Delhi-110002, India

**Keywords:** Anterior tibial artery, pseudoaneurysm, Steinmann pin

## Abstract

An anterior tibial artery pseudoaneurysm is a rare and unexpected complication of Steinmann pin insertion. We describe the case of an 18-year-old boy, who sustained such an injury to the anterior tibial artery during this procedure. Diagnosis was confirmed on a magnetic resonance (MR) angiogram. Aneurysmal sac excision with lateral repair of the vessel wall was performed. Postoperatively, a good flow was documented on a follow-up MR angiogram. This case highlights a major and unexpected complication of a so-called minor procedure. Too posterior a pin placement in the proximal tibia should be avoided to prevent such injuries.

## INTRODUCTION

Proximal tibial pin insertion is a common orthopedic procedure. Apart from common complications like pin tract infection and pin loosening, serious complications like common / deep peroneal nerve paralysis leading to foot drop is also known.^1^ We report our experience of managing a case of pseudoaneurysm of the anterior tibial artery resulting from proximal tibial pin insertion, which to the best of our knowledge and search of literature has not been previously reported in English language literature.

## CASE REPORT

**Figure 1:**
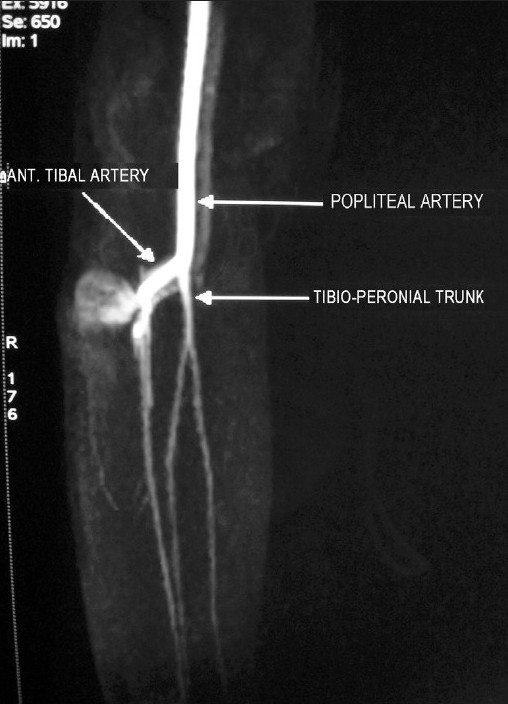
Preoperative MR angiogram showing the pseudoaneurysm arising from the anterior tibial artery

**Figure 2:**
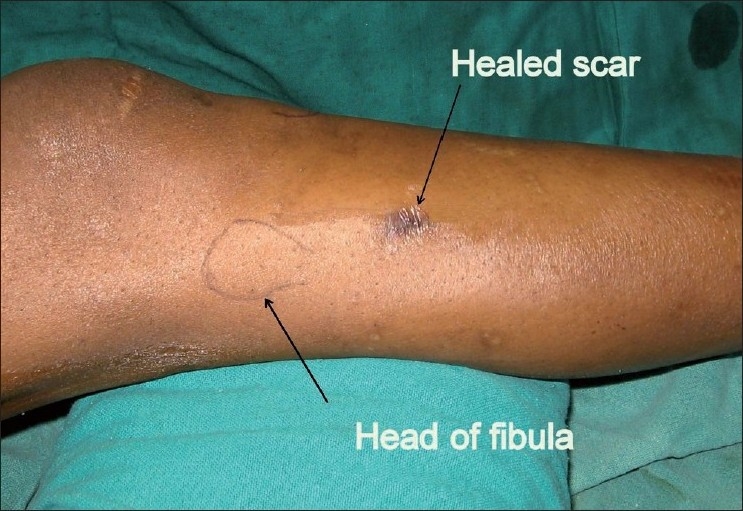
Clinical photograph showing the scar of the upper tibial pin

An 18-year-old boy presented with a swelling in the right proximal leg following skeletal traction by a Steinmann pin, for the treatment of central fracture dislocation of the hip at a different hospital. The referral notes revealed a history of continuous and profuse bleeding from the pin tract site. On removal of the tibial pin the patient had repeated bouts of profuse bleeding from the wound. Each episode of bleeding had to be controlled with pressure bandaging leading finally to healing of the skin wound. At this time, the patient noticed a gradually increasing swelling in the vicinity of the healed pin-track. An MR angiogram was done at the index center, which revealed a pseudoaneurysm originating from the proximal portion of the anterior tibial artery [Figure 1]. The patient was then referred to us for further management. On examination, an ill-defined, fluctuant, compressible, nontender swelling was noted on the anterolateral aspect of right proximal leg. A healed scar mark of an upper tibial pin was noted [Figure 2]. The swelling was pulsatile and a bruit was audible. Dorsalis pedis and posterior tibial pulses were palpable. There was no distal neurological deficit. The clinical diagnosis was that of a pseudoaneurysm.

Doppler ultrasound examination revealed a 3 cm × 3 cm soft mass with a hypoechoic oval lumen, the fluid filling consistent with the arterial flow.

**Figure 3:**
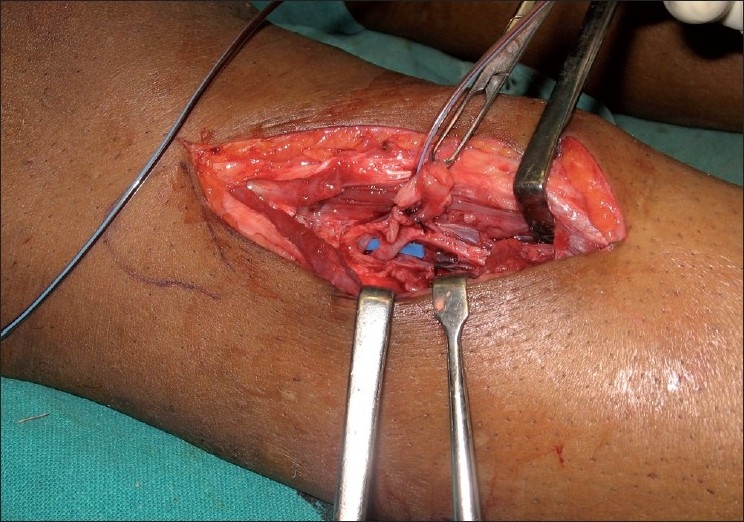
Exposure of the anterior tibial artery with a pseudoaneurysm sac for proximal and distal control

**Figure 4:**
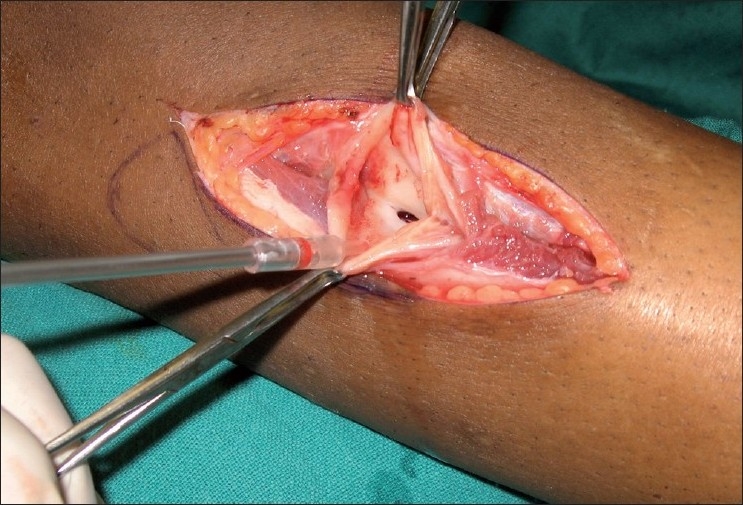
Communication of the pseudoaneurysm sac with the main arterial lumen

**Figure 5:**
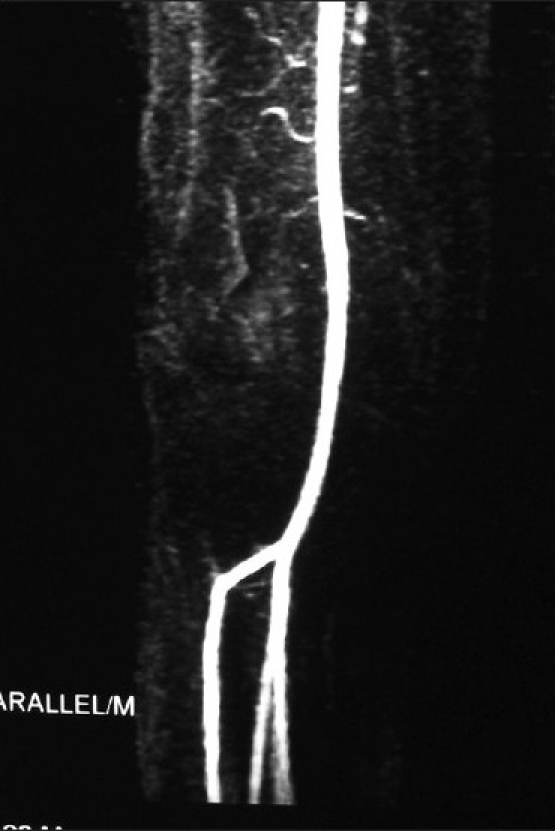
Follow-up MR angiogram at three weeks, showing a good cross-sectional flow across the site of anastomosis

Under epidural anesthesia and a tourniquet, the pseudoaneurysm was exposed through a longitudinal incision over the swelling. Proximal and distal control of the anterior tibial artery was obtained [Figure 3]. The aneurysmal sac was incised and the clot was removed to reveal a partial transection 3 mm × 3 mm in size, through which the sac was communicating with the arterial lumen [Figure 4]. The sac was excised and the rent in the artery was repaired by a lateral suture with 8-O nylon, using loupe magnification. The epidural catheter was retained for 48 hours for sympathetic blockade and the patient was administered Tab aspirin 100 mg OD and Tab pentoxiphylline therapy for four weeks. A follow-up MR angiogram at three weeks revealed good cross-sectional flow at the site of repair [Figure 5].

## DISCUSSION

**Figure 6:**
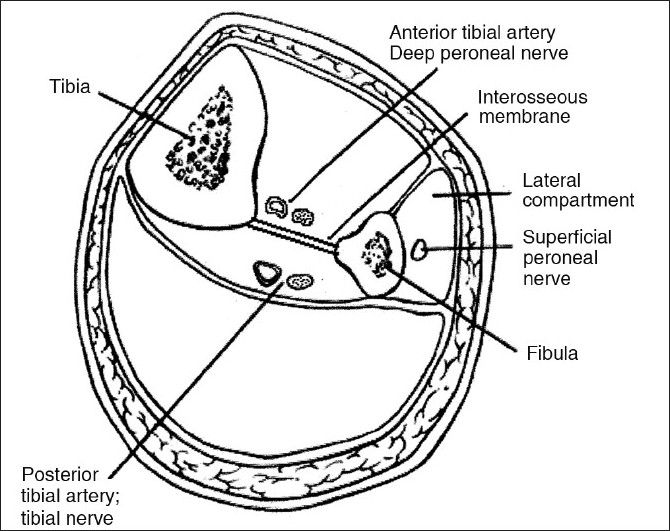
Cross-section of the leg showing the relationship of the anterior tibial artery with the tibia and intraosseous membrane

The proximal tibial Steinman pin is routinely inserted for skeletal traction, for hip and thigh fractures. The recommended site of insertion is a point 2 cm distal and posterior to the tibial tuberosity, directed lateral to medial.^2^ Excessive anterior insertion of the pin increases the chances of the pin cutting through the anterior cortex of the tibia. A misdirected pin is known to injure the deep peroneal nerve.^1^ To the best of our knowledge, this procedure has not been reported to have caused anterior tibial artery injury. The anterior tibial artery is a branch of the popliteal artery that arises at the distal border of the popliteus muscle. It passes anteriorly and lies on the interosseous membrane, which in turn is attached to the posterolateral border of the tibia [Figure 6]. The artery is relatively fixed here, as it just enters the anterior compartment after hooking across the proximal border of the intraosseous membrane.^3^ The tibia is triangular in cross-section and it has been recommended that pins be inserted in its posterior half for better purchase, especially for external skeletal fixation. This case therefore highlights the vulnerability of the anterior tibial artery to become injured by an excessive posterior pin insertion in the proximal tibia.

The time from injury to the detection of the pseudoaneurysm has been reported to vary from hours to years, depending on the site, size, and clinical signs and symptoms.^4^ The latter may include pain, swelling, a pulsatile mass, an audible bruit, distal emboli, blood oozing from the operative site, and neurological deficit. They may mimic soft tissue masses including abscesses, neoplasms or ganglions.^5^–^8^ Inadvertent surgery in such cases may lead to uncontrolled hemorrhage. Therefore, a high index of suspicion is justified in dealing with such swellings in the vicinity of a major vessel.^9^,^10^

Surgical correction of a false aneurysm should be performed as soon as possible after the diagnosis is made, to prevent the complications of rupture or rapid expansion with resultant pressure on the adjacent nerves.^10^,^11^ Urgent surgery is advocated if neurological symptoms develop.^7^

Several methods of repair have been described in the literature for the treatment of a pseudoaneurysm.^7^,^10^,^12^,^13^ These include excision of the aneurysmal sac and repair of the lateral wall, endoaneurysmorrhaphy — obliterative or reconstructive, limited arterial resection, and end-to-end anastomosis, resection, and graft placement and ligation of the artery (if non-critical), for example, radial artery and distal posterior tibial artery.

It has been suggested that pseudoaneurysm of the anterior tibial artery can be managed by ligation, provided the posterior tibial artery is patent and maintains vascularity to the anterolateral compartment and distal limb.^11^ However, this may produce symptoms of chronic exertional compartment syndrome in high-demand patients. Therefore, reconstruction / repair of the injured blood vessel, even in double vessel situations is desirable. The method of reconstruction depends upon the size of the communication between the injured vessel and the aneurysm. A large size demands endoaneurysmorrhaphy, while a small opening can be repaired by a lateral repair, as in this case. Although the lateral suture technique is supposed to create a narrowing at the site of repair, the use of 8-O sutures under magnification in our case created a near normal cross-sectional flow across the site of the anastomosis.

To conclude, the present case highlights the possibility of a major complication (anterior tibial artery injury) resulting from a so-called minor surgical procedure (Steinmann pin insertion). It may therefore be prudent to have a mid cross-sectional insertion of pins in the tibia instead of posterior, proving yet again that the middle path is probably the most appropriate.
